# Makorin Ring Finger Protein 1 as Adjunctive Marker in Liquid-based Cervical Cytology

**DOI:** 10.1097/MD.0000000000002425

**Published:** 2016-01-22

**Authors:** Maria Lee, Min Young Chang, Ha-Yeon Shin, Eunah Shin, Sun Won Hong, Kyung-Mi Kim, Doo Byung Chay, Hanbyoul Cho, Jae-Hoon Kim

**Affiliations:** From the Department of Obstetrics and Gynecology, Seoul National University College of Medicine (ML), Department of Obstetrics and Gynecology, Gangnam Severance Hospital, Yonsei University College of Medicine (MYC, H-YS, DBC, HBC, J-HK), Department of Pathology, CHA Gangnam Medical Center, CHA University (ES), Department of Pathology, Yonsei University College of Medicine (SWH); and Department of Pathology, Samsung Medical Center, Sungkyunkwan University School of Medicine, Seoul, South Korea (K-MK).

## Abstract

To assess the utility of makorin ring finger protein 1 (MKRN1) as a marker of cervical pathology.

A PROspective specimen collection and retrospective Blinded Evaluation study was conducted. Liquid-based cytology samples were collected from 187 women, embedding all residuals as cell blocks for immunohistochemical staining of MKRN1 and P16 ^INK4a^. Results of liquid-based cervical cytology, immunostained cell block sections, and human papillomavirus (HPV) hybrid capture (with real-time polymerase chain reaction) were analyzed. Clinical outcomes were analyzed overall and in subsets of specimens yielding atypical squamous cells of undetermined significance or low-grade squamous intraepithelial lesions.

Makorin ring finger protein 1 positivity and grades (1–3) of cervical intraepithelial neoplasia (CIN) increased in tandem (CIN1, 32.4%; CIN2, 60.0%; and CIN3, 80.0%), reaching 92.3% in invasive cancer. Sensitivity, specificity, positive predictive value, and negative predictive value in detecting CIN2+ via MKRN1 were 73.8%, 76.8%, 75.6%, and 75.0%, respectively. The performance of liquid-based cytology was poorer by comparison (61.3%, 69.5%, 66.2%, and 64.8%, respectively), and HPV assay (versus MKRN1 immunohistochemical staining) displayed lower specificity (67.7%). Combined HPV + MKRN1 testing proved highest in sensitivity, specificity, positive predictive value, and negative predictive value (71.8%, 85.5%, 82.3%, and 76.5%, respectively), whereas corresponding values for cytology + HPV (60.6%, 81.8%, 75.4%, and 69.2%) and cytology + MKRN1 (58.8%, 84.1%, 78.3%, and 67.7%) were all similar. In instances of atypical squamous cells of undetermined significance or low-grade squamous intraepithelial lesions, the HPV + MKRN1 combination performed best by above measures (100%, 72.7%, 73.9%, and 100%), followed by cytology + MKRN1 (100%, 50.0%, 60.7%, and 100%).

Makorin ring finger protein 1 displayed greater sensitivity and specificity than liquid-based cytology and proved more specific than HPV assay. In combination testing, MKRN1 + HPV showed the highest sensitivity and specificity levels. The MKRN1 biomarker may be a useful adjunct in primary cervical cytology screening.

## INTRODUCTION

Regimented cytologic screening has contributed significantly to reducing the incidence of cervical cancer,^1^ but as the sole means of testing, its low diagnostic accuracy (owing to limited reproducibility in cervical intraepithelial neoplasia [CIN] detection) is problematic.^2^ Consequently, novel molecular assays have emerged to augment this conventional approach.^3^

Given that human papilloma virus (HPV) infection is implicated in cervical carcinogenesis, a recent study has found HPV deoxyribonucleic acid (DNA) testing more sensitive than cervical cytology, detecting high-grade CIN earlier^4^ and thus furthering efforts to prevent invasive cancer. Unfortunately, HPV screening has low specificity in this setting. Although such infections are common and are apt to resolve naturally within 1 to 2 years, both transient bouts and persistent infections (accounting for high-grade CIN)^4^ yield positive test results.

Many researchers are now focused on developing more effective screening tests for CIN detection, hoping to improve the specificity of cytologic preparations and HPV tests while maintaining the generally high respective sensitivities. Biomarkers of HPV-related genes strongly expressed in carcinogenesis are of particular interest. Prime examples include Ki-67 (involved in cellular proliferation) and p16^INK4a^ (a cell-cycle regulatory protein), both of which have been identified in prior studies as markers of CIN.^4–8^ Conversely, p16^INK4a^ immunoreactivity is also observed in endocervical or metaplastic cells and in benign atrophic cells, requiring attention to cell morphology when interpreting stained specimens.^9^

Makorin ring finger protein 1 (MKRN1) is a transcriptional coregulator, an E3 ligase, and a negative regulator of tumor suppressor genes p53 and p21. It has been noted that a reduction in MKRN1 induces growth arrest by activating p53 and p21.^10^ Makorin ring finger protein 1 also blocks cancer cell death by inducing ubiquitination and thus promoting degradation of Fas-associated protein with death domain, a key element in death receptor-activated extrinsic apoptosis. Because MKRN1 and Fas-associated protein with death domain participate in necrosome formation and necroptosis regulation, downregulation of MKRN1 understandably has been shown to have a major inhibitory effect on tumor enlargement in breast cancer. Furthermore, MKRN1 messenger ribonucleic acid levels are significantly higher in cancerous than in normal cervical tissue.^11^

The current study was conducted to determine if MKRN1 immunohistochemical (IHC) staining is a viable adjunct in diagnosing cervical cancer or its precursor lesions. Specifically, 4 diagnostic procedures (cytology, HPV assay, MKRN1, and p16^INK4a^ immunostaining) were evaluated in terms of sensitivity, specificity, positive predictive value (PPV), negative predictive value (NPV), and accuracy in diagnosing CIN2+. For this purpose, residual liquid-based cytology samples enabled immunostaining of MKRN1 and p16^INK4a^ biomarkers.

## METHODS

### Study Population

Specimens were prospectively collected between July, 2013 and February, 2014, following approval by the Institutional Review Board for Clinical Research at Gangnam Severance Hospital. The study population (n = 189) consisted of women ≥18 years old who were referred to the above facility for abnormal cervical cytology results; who were admitted with benign conditions (eg, uterine fibroids or adenomyosis) for hysterectomies; or who visited the hospital for regular checkups and routine cervical cytology screening. Each enrolee was subjected to cervical cytology screening, HPV assay, and immunostaining for MKRN1 and p16^INK4a^ markers (per protocol). Exclusions were as follows: age <18 years, prior hysterectomy, previous cancer of noncervical origin, treatment for CIN or invasive cancer within last 5 years, chronic illness with immunocompromise, or refusal to consent/participate. The clinical performance of each test method was determined retrospectively, based on histologic findings in punch biopsy or hysterectomy specimens.

### Liquid-based Cervical Cytology

A liquid-based cervical cytology sample was obtained from each patient via Cytobrush device (ThinPrep 2000 system, Cytyc Corp, Boxborough, MA). Two pathology specialists (same institution affiliates) rendered all diagnoses, based on the Bethesda system (2001). In the event of conflicting results (primary care records versus referral hospital), higher grade lesions prevailed for analysis.

### Human Papillomavirus Testing

The Hybrid Capture 2 (HC2) assay (Digene Corp, Gaithersburg, MD), which is designed to detect 13 types of high-risk HPV, was routinely performed. Results were expressed as a ratio between light emitted from a test specimen and from 1 pg/mL HPV DNA (average of 3 control specimens). For example, 1 relative light unit would correspond with 1 pg/mL HPV DNA in a specimen tested. Because HC2 assays cannot distinguish between single and multiple infections, nor distinguish between HPV types 16 and 18, the cobas 4800 HPV test (Roche, Pleasanton, CA) was also performed. Results were reported as negative, type 16 or 18, and other types. Positive HPV testing was equated with HPV positivity by HC2 assay or detection of any HPV type (HPV 16 or 18 and others).

### Histology

Punch biopsies were obtained by a specialist in colposcopy to confirm normal cytology results or corroborate the presence of high-risk HPV infection. Patients with CIN1 or lower grades were followed without further treatment. In every patient with CIN3+ and in most with CIN2, either a loop electrosurgical excision procedure or a hysterectomy was done. Those diagnosed with invasive cervical cancer underwent radical hysterectomy. If punch biopsy and loop electrosurgical excision procedure or hysterectomy specimens resulted in conflicting diagnoses, the highest grade prevailed for analysis.

### Cell Block Preparation

Cell blocks for immunostaining of MKRN1 and p16^INK4a^ were prepared from residual samples (ThinPrep Pap Test, Hologic Inc, Marlborough, MA), based on a method described in a number of previous studies. Specifically, residual PreservCyt Solution (Cytyc) was centrifuged (1500 rpm, 5 minutes), discarding the supernatant and resuspending the pellet in 5 to 8 drops of plasma. Next, 2% calcium chloride was added, mixed well, and left to coagulate (20 minutes). The resultant clot was wrapped in lens paper, placed in a tissue cassette, and fixed (10 minutes) in 10% neutral buffered formalin. Completed preparations were sectioned at 4.0 μm and stained with hematoxylin and eosin. Cellular morphology and degree of preservation were assessed.

### Makorin Ring Finger Protein 1 Immunohistochemical Staining

**FIGURE 1:**
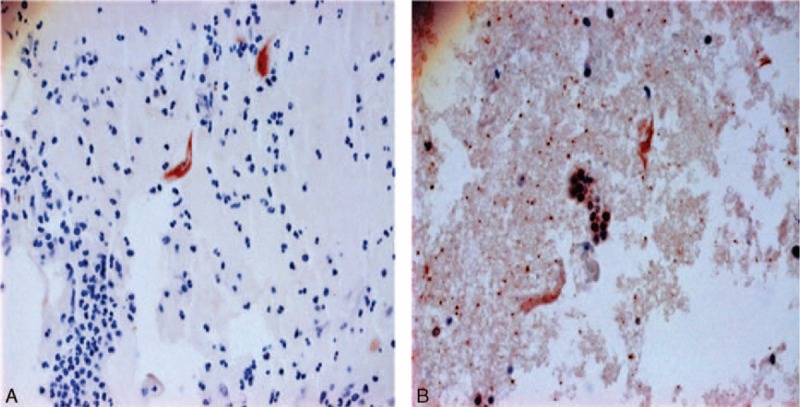
Immunohistochemical staining of makorin ring finger protein in representative sections of normal cervical epithelium (A) and high-grade cervical intraepithelial neoplasia (B).

To determine MKRN1 expression, prepared cell blocks were again sectioned at 400 μm, and IHC staining was performed using rabbit anti-MKRN1 antibody and affinity purified makorin-1 antibody (Bethyl Laboratories Inc, Montgomery, TX). The UltraVision LP Detection System kit (Thermo Fisher Scientific, Fremont, CA) was used for indirect staining, using a biotin-linked secondary antibody labelled with horseradish peroxidase. The peroxidase reacts with 3,3′-diaminobenzidine tetrahydrochloride, catalyzing a color change, and the slides are counterstained with hematoxylin. Sections were interpreted by a pathology specialist, equating a positive result with visible nuclear staining (Figure 1).

### P16^INK4a^ Immunohistochemistry

Expression of p16^INK4a^ in cell block sections was determined using a p16^INK4a^-specific monoclonal antibody (Clone E6H4, Santa Cruz Biotechnology Inc, Dallas, TX) and CINtec p16-^INK4a^ Cytology kit (Dako Cytomation [now Dako A/S], Glostrup, Denmark). The slides were then incubated (30 minutes) with secondary goat anti-mouse antibody (Dako), followed by 3,3′-diaminobenzidine tetrahydrochloride (Dako) incubation (10 minutes) and finally counterstaining with Mayer hematoxylin.

### Statistical Analysis

In diagnosing CIN2+, sensitivity, specificity, PPV, and NPV were calculated for each test method (cytology, HPV test, and IHC staining of MKRN1 and p16^INK4a^) using standard software (SAS v9.2, SAS Institute Inc, Cary, NC). Based on calculated sensitivity and specificity values, diagnostic accuracy of each method was determined from receiver operating characteristic curves. Identical analyses were performed for each 2-method combination tested in the entire cohort and in patient subsets showing atypical squamous cells of undetermined significance (ASCUS) or low-grade squamous intraepithelial lesions (LSIL). Each result was expressed as mean, with a 95% confidence interval. Statistical significance was set at *P* < 0.05.

## RESULTS

### Cytology Versus Histology

Results of cytology and histologic examinations are shown in Table 1. Of the 189 patients originally enrolled for study, 187 remained for the final analysis (excluding 2 for failure to procure cervical cells). Ultimately, 47 (25.1%) normal and 140 (74.9%) abnormal cytology results were recorded. In the abnormal subset, histologic examination confirmed 8 (17%) with CIN1, 3 (6.4%) with CIN2, 3 (6.4%) with CIN3/cervical carcinoma in situ (CIS), and 1 (2.1%) with invasive cancer. In patients (n = 140) with abnormal cytology results, ASCUS or atypical glandular cells of undetermined significance were evident in 22, including 5 (22.7%) with negative histologic presentations, 4 (18.2%) occurrences of CIN2, 6 (27.3%) instances of CIN3/CIS, and 2 patients (9.1%) with invasive cancer. In patients (n = 55) with LSIL, histologic examinations were normal in 17, whereas 20 (36.4%) were diagnosed as CIN1, 12 (21.8%) as CIN2, and 6 (10.9%) as CIN3/CIS. In patients (n = 57) demonstrating atypical squamous cells on cytology (high-grade squamous intraepithelial lesion/high-grade squamous intraepithelial lesion not excluded), CIN1 was evident histologically in 5 (8.8%), CIN2 in 13 (22.8%), and CIN3/CIS in 31 (54.4%), whereas 6 (10.5%) showed invasive cancer. In patients (n = 6) diagnosed with cancer by cytology, CIN3/CIS was documented in 1 (16.7%), and 5 (83.3%) showed invasive cancer.

**TABLE 1:**
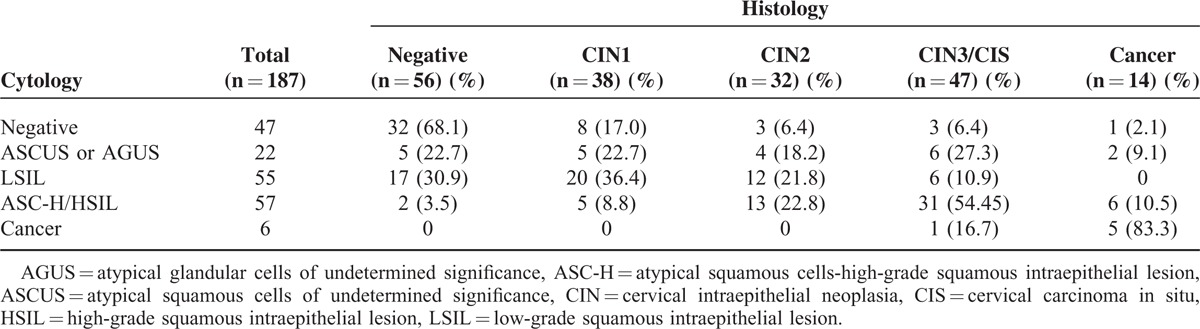
Comparison of Liquid-based Cervical Cytology and Tissue Histology

### Test Performances Versus Histology

Performance levels of the 4 cervical cancer screening tests under investigation are shown by histologic diagnoses in Table 2. Findings of reactive cellular change or chronic nonspecific inflammation on cytology were considered negative, with ASCUS or higher grades of CIN qualifying as positive results. In 56 patients (29.9%), no pathology was evident, including 24 (42.9%) who initially appeared positive on cytology and 21 (37.5%) who tested positive for the p16^INK4a^ marker. Patient totals in those testing positive by HPV assay and MKRN1 immunostaining were 10 (17.9%) and 12 (21.4%), respectively. Of 38 patients with CIN1, 30 showed positivity by cytology, HPV assay, and p16^INK4a^ immunostaining, whereas 12 tested positive for MKRN1. Of 32 patients (17.1%) diagnosed with CIN2, 29 (90.6%), were positive by cytology and HPV assay, with similar rates for MKRN1 and p16^INK4a^ (20/32 [62.5%] and 21 [65.6%], respectively). In 47 patients (25.1%) with CIN3/CIS, positive results were recorded as follows: cytology, 44 (93.6%); HPV assay, 46 (97.9%); MKRN1, 35 (74.5%); and p16^INK4a^, 36 (76.6%). In 14 patients (7.5%) with invasive cancer, positivity totals for cytology, HPV test, and MKRN1 marker were the same at 13 (92.9%), with 12 (85.7%) positive results for p16^INK4a^.

**TABLE 2:**
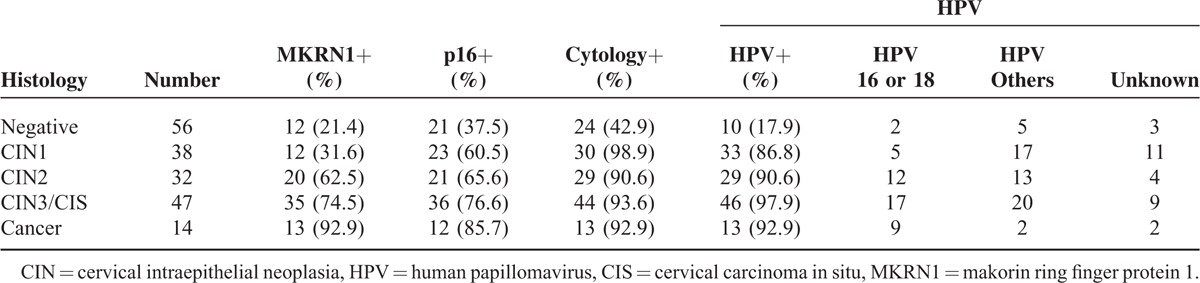
Distribution of Tissue Histology by Test Method (Makorin Ring Finger Protein 1, P16^INK4a^, Cytology, and Human Papillomavirus Serotypes)

### Performance of Clinical Test Methods in Detecting Cervical Intraepithelial Neoplasia 2+

The goal of this study was to accurately diagnose precancerous cervical pathology. To determine the diagnostic accuracy of the 4 screening tests, we calculated sensitivity, specificity, PPV, NPV, and diagnostic accuracy for both individual tests and 2-method combinations (Table 3). Based on histology, CIN2+ constituted disease positivity. Identical analyses were conducted for subgroups of patients showing ASCUS or LSIL on cytology (Table 4). In terms of individual tests, the HPV assay displayed the highest sensitivity rate (94.6%), but its specificity was low (54.3%). Liquid-based cytology, the current primary screening method, showed sensitivity (92.5%) similar to HPV assay, but had the lowest specificity (42.6%) of the 4 test methods. Makorin ring finger protein 1 immunostaining proved less sensitive than cytology (73.1% versus 92.5%) but more specific than HPV assay (74.5% versus 54.3%). Compared with the MKRN1 marker, immunostaining of P16^INK4a^ proved similar in sensitivity (74.2% versus 73.1%) but lower in specificity (53.2% versus 74.5%). Used in combination, cytology and HPV assay (cytology + HPV) showed the highest sensitivity (90.3%) but the lowest specificity (66.0%) of all combinations studied. Combined use of HPV assay and MKRN1 immunostaining (HPV + MKRN1) showed the highest specificity (87.2%). Equivalent sensitivity (71.0%) and specificity (77.7%), however, were observed for cytology and MKRN1 marker (cytology + MKRN1) or HPV assay and p16^INK4a^ marker (HPV + p16^INK4a^) in combination. The second lowest specificity (69.1%) was encountered with cytology and p16^INK4a^ marker (cytology + p16^INK4a^) in combination, with cytology + HPV showing the lowest specificity. Combined marker screening (MKRN1 + p16^INK4a^) had the second highest specificity (80.9%) but the lowest sensitivity (62.4%). The 2-method combination with the highest specificity was HPV + MKRN1. In terms of diagnostic accuracy, HPV assay and MKRN1 immunostaining were the most accurate methods in single use (74.3% and 73.8%, respectively), whereas 2-method accuracy was highest (78.1%) for cytology + HPV and HPV + MKRN1.

**TABLE 3:**
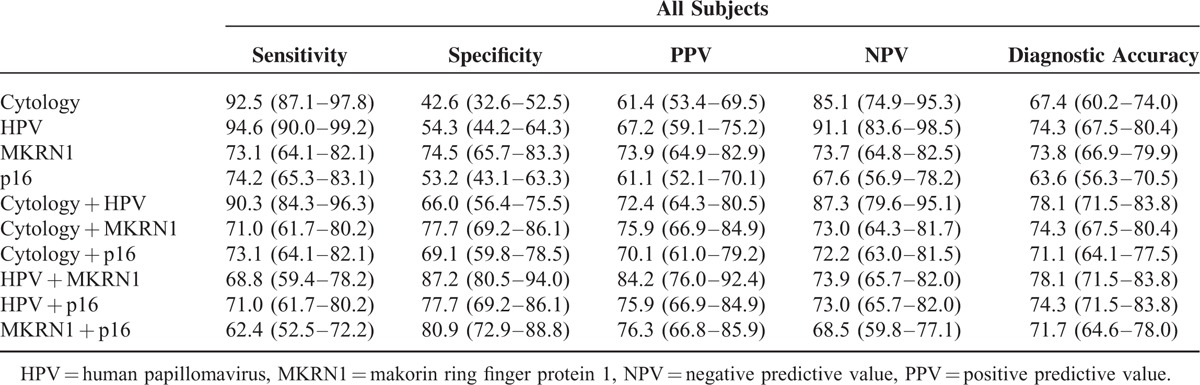
Performances of Conventional Cytology, Human Papillomavirus Assay, and Biomarker (Makorin Ring Finger Protein 1 and p16^INK4a^) Immunostains in Detecting Cervical Intraepithelial Neoplasia 2+ Overall (All Subjects)

**TABLE 4:**
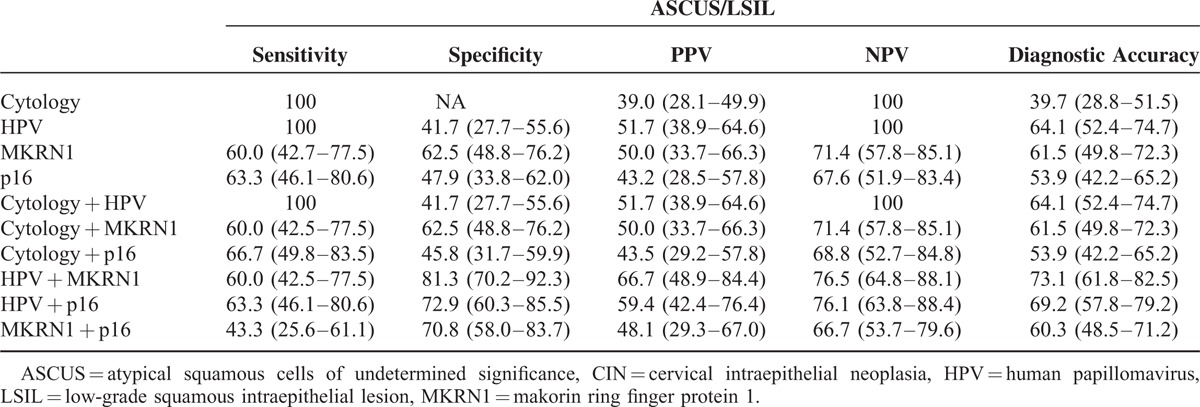
Performances of Conventional Cytology, Human Papillomavirus Assay, and Biomarker (Makorin Ring Finger Protein 1 and p16^INK4a^) Immunostains in Detecting Cervical Intraepithelial Neoplasia 2+ Within Atypical Squamous Cells of Undetermined Significance /Low-grade Squamous Intraepithelial Lesion Patient Subsets

In patients with ASCUS or LSIL patients, cytology and HPV assay again showed the highest individual sensitivity (100%), with low specificity. Sensitivities of MKRN1 and p16^INK4a^ markers were similar (60.0% and 63.3%, respectively), but MKRN1 showed the highest specificity (62.5%). In combination, cytology + HPV earned the highest sensitivity (100%) but the lowest specificity (41.7%), whereas HPV + MKRN1 showed high specificity at 81.3%. As individual tests, HPV assay and MKRN1 immunostaining were the most accurate (64.1% and 61.5%, respectively), as with patients overall, whereas cytology was least accurate (39.7%). Of the 2-method combinations analyzed, HPV + MKRN1 showed the highest diagnostic accuracy (73.1%), surpassing that of cytology + HPV (64.1%).

## DISCUSSION

To compensate for the low specificities of cytology and HPV testing, researchers have focused on p16^INK4a^ as a novel marker for cervical neoplasia. In a large-scale randomized trial, targeting of p16^INK4a^ reportedly increased diagnostic specificity and correlated strongly with histologic results.^7^ Assessment of p16^INK4a^ overexpression (via immunostaining) and HPV assay in combination yielded better diagnostic accuracy than conventional cervical cytology and HPV testing. Consequently, the pursuit of useful adjuncts to existing methods is still a high priority in ongoing research. In a study conducted by Lee et al^11^, overexpression of the MKRN1 protein correlated with cervical cancer in vivo. Another previous study has also shown that MKRN1 expression levels in cervical tissue are proportional to tumor stage or grade of tumor, correlating positively with pAKT and negatively with phosphatase and tensin homolog (PTEN) expression levels.^12^

Outcomes of the current study indicate that HPV and MKRN1 determinations are similar in terms of diagnostic accuracy (∼74%), each surpassing cervical cytology (67.4%). In dual applications (cytology + HPV and HPV + MKRN1), the diagnostic accuracies, however, were comparable (78.1%) while specificities differed. The combination of cytology + HPV had the lowest specificity, but individual sensitivities were highest (92.5% and 94.6%, respectively). Thus, the MKRN1 marker essentially complements the low-specificity, high-sensitivity nature of HPV assay, raising overall specificity from 54.3% to 87.2% and delivering the highest diagnostic accuracy. Hence, the benefit conferred justifies the effort and cost of an added test.

The most difficult scenarios in cervical cancer screening are those complicated by ASCUS and LSIL. Previous studies contend that such changes will likely disappear^7^; therefore, unnecessary procedures may be avoided by conservative measures (ie, follow-up observation only). Nonetheless, it is alarming that a high percentage of patients (55/77, 71.4%) in our cohort with these cytologic abnormalities eventually were diagnosed as CIN1+, and CIN2+ was confirmed histologically in 30 (39.0%) of these patients. This suggests a clear propensity for later development of invasive cancer, making it imperative to separate those who require active intervention from others for whom observation is sufficient. As such, we investigated the clinical performance of screening tests in patient subsets showing ASCUS or LSIL on cytology. Outcomes were similar to those observed in patients overall. In combined HPV + MKRN1 testing, specificity was nearly 2-fold that of cytology + HPV (81.3% versus 41.7%), resulting in the highest diagnostic accuracy (76.5%).

According to epidemiologic studies of HPV, infection rates are higher in sexually active women <35 years of age, although most episodes resolve naturally.^13^ Because younger women typically have plans of future childbirth, the increased likelihood of procedure-related obstetric morbidity, such as preterm birth or incompetent internal os, may become important.^14^ Moreover, cytology results indicative of ASCUS, LSIL, or HPV infection are a potential source of psychologic stress in women. Therefore, a physician's decision to opt for follow-up observation (with patient reassurance) or halt progression to invasive cancer through aggressive testing and treatment is critical. The exceptional specificity of HPV + MKRN1 in patients of questionable status (ASCUS or LSIL) is particularly noteworthy.

Human papillomavirus vaccination coverage has increased significantly after approximately 8 years of usage for cervical cancer. The US Food and Drug Administration granted approval of Gardasil(Merck & Co., Inc., Whitehouse Station, NJ USA) in 2006 and Cervarix(GlaxoSmithKline Biologicals, Rixensart, Belgium) in 2009.^15^ As the proportion of patients with invasive cancer or rapidly progressive high-grade CIN (transitioning to invasive cancer) declines, owing to effective curtailment of HPV 16 and 18 serotypes, a commensurate decline in the PPV of cytology is expected. Thus, the preferred test for primary cervical cancer screening is gradually shifting from cytology to the HPV DNA assay, despite the low specificity. Makorin ring finger protein 1 may then play a key adjunctive role in cervical screening, as demonstrated herein.

Cytology screening relies on subjective assessments that prove disproportionately unsatisfactory in less skilled hands. Trained professionals must carefully search for atypical cells, which may be obscured by a dearth of normal epithelial elements. Moreover, intra- or interobserver discrepancies are possible in interpreting ASCUS and high-grade squamous intraepithelial lesion. A systematic review of 62 studies conducted by Fahey et al^16^ to determine the accuracy of the Pap tests showed wide variations in sensitivity (11%–99%) and specificity (14%–97%). In contrast, HPV assay is an objective and highly sensitive automated procedure, with limited opportunity for medicolegal claims because of disputed results.

The current study is the first to report the features and benefits of MKRN1 as a diagnostic marker for invasive cervical cancer/CIN. This study was free of selection bias, given prospective collection of specimens and treatment guidelines developed after the study was planned. All data were analyzed retrospectively, based on histology reports. Makorin ring finger protein 1 IHC staining was found to be an effective test method that complemented the low specificity of cytology and HPV assay. Residual specimens from liquid-based cytology or cobas HPV analysis may be used for staining, without the added effort, discomfort, or anxiety during gynecologic examinations. Of particular importance, this method may curb unnecessary colposcopically directed biopsy and referral rates, thereby reducing medical costs while safely extending screening intervals.

In terms of future study considerations, the relationship between clinical performance of HPV + MKRN1 and patient age should be investigated, assessing study population subsets. In addition, polymerase chain reaction-based experiments are needed to confirm the possibility of more convenient method. Above all, cost-effective analysis is required to clarify the best screening strategy including MKRN1 IHC method for diagnosing CIN2+. Follow-up analysis of whether MKRN1 presence or absence impacts progression to high-grade CIN is another important issue.

## CONCLUSIONS

In conclusion, comparative clinical performances of MKRN1 IHC staining and currently available cervical cancer screening tests indicate that HPV + MKRN1 is the ideal test combination, despite equivalent accuracy of cytology + HPV. The MKRN1 and HPV methods complement 1 another, considering the high sensitivity of HPV assay and the high specificity of the MKRN1 biomarker. Furthermore, the HPV + MKRN1 combination was most accurate for diagnosing CIN2+ in ASCUS/LSIL patient subsets. We anticipate that MKRN1 IHC staining will ultimately emerge as a useful adjunct to routine cervical cancer screening.
